# Phenomenology and content of the inhaled *N*, *N*-dimethyltryptamine (*N*, *N*-DMT) experience

**DOI:** 10.1038/s41598-022-11999-8

**Published:** 2022-05-24

**Authors:** David Wyndham Lawrence, Robin Carhart-Harris, Roland Griffiths, Christopher Timmermann

**Affiliations:** 1 Department of Family & Community Medicine, Faculty of Medicine, University of Toronto, Toronto, Canada; 2 Mount Sinai Hospital, Sinai Health System, 600 University Avenue, Toronto, M5G 1X5 Canada; 3 Faculty of Kinesiology & Physical Education, University of Toronto, Toronto, Canada; 4 Centre for Psychedelic Research, Division of Psychiatry, Department of Brain Sciences, Imperial College London, London, UK; 5 Psychedelics Division, Neuroscape, Department of Neurology, University of California, San Francisco, CA USA; 6 Departments of Psychiatry and Neuroscience, Center for Psychedelic and Consciousness Research, Johns Hopkins University School of Medicine, Baltimore, MD USA

**Keywords:** Neuroscience, Psychology, Neurology

## Abstract

Understanding the phenomenology and content of the inhaled *N*, *N*, dimethyltryptamine (*N*, *N*-DMT) experience is critical to facilitate and support ongoing research and therapeutic models targeting mental health conditions and central nervous system pathology. A qualitative analysis was conducted of all *N*, *N*-DMT experiences posted to the r/DMT Reddit community over a 10-year period from 2009 to 2018. A total of 3778 experiences from 3305 posts were included in this study. A median dose of *N*, *N*-DMT of 40.0 mg [interquartile range (IQR), 27.5 to 50.0] and a median experience duration of 10 min (IQR, 5.0 to 15.0) were identified. The most common somatic effects were somaesthesias (n = 1415, 37.5%) and an auditory ringing (n = 583, 15.4%). Visualizations predominantly consisted of fractals, shapes, patterns (n = 1231, 32.6%) and vivid colours (n = 953, 25.2%). Entity encounters were reported in 45.5% (n = 1719) of the experiences and involved predominantly a feminine phenotype (n = 416, 24.2%); deities (n = 293, 17.0%); aliens (n = 281, 16.3%); creature-based entities (n = 158, 9.2%, including reptilian and insectoid beings); mythological beings (n = 144, 8.4%, including machine elves); and jesters (n = 112, 6.5%). Entity interactions were predominantly positive (n = 600, 34.9% of encounters) involving benevolent, comforting, protecting, or outwardly caring interactions. A companion-type, pedagogical, or guide-type interaction was identified 32.4% of encounters (n = 557). Common typology, architecture, and structural features of the “DMT world” included descriptions of alternate or higher dimensions (n = 952, 25.2%); rooms [n = 582, 15.4%, including the “waiting room” (n = 105, 2.8%)], and a tunnel (n = 390, 10.3%). Features of mystical and ego-dissolution experiences were common. Additional rewarding aspects were identified, including a sense of familiarity and the acceptance/removal of the fear of death. Challenging and difficult responses were less frequent but also documented. Statements of profundity were identified in 232 experiences (6.1%), including pronouncing the experience or an aspect of the experience as the most “beautiful” or feeling the most “beautiful” of their life (n = 47, 1.2%). This study identified common phenomenological themes and content of naturalistic inhaled *N*, *N*-DMT experiences. Major thematic domains included (1) physical and somatic experiences; (2) visualizations and imagery; (3) entity encounters including entity phenotype, descriptors, attributes, disposition, and characteristics of the interaction; (4) typology, architectural features, structural characteristics, and scenery of the “DMT world”; (5) alerations in consciousness (including mystical experiences, out-of-body experiences, and ego-dissolution); (6) emotional responses (including positive, rewarding, difficult, and challenging); and (7) statements of profundity.

## Introduction

*N*, *N***-**Dimethyltryptamine (*N*, *N*-DMT) is a naturally occurring classic psychedelic with psychoactive properties that are mediated primarily via the serotonergic pathway and serotonin 2A (5-HT_2A_) receptor agonism^1^. Plant-based DMT has been used for centuries for ritual healing ceremonies and spiritual practices^2–5^. Early reports of DMT-use derive from indigenous communities in the Amazon-basin and involve the oral consumption of *Ayahuasca*^3,4,6–8^, which is a Quechua term translated to “vine of the souls” or “vine of the dead”^9^. *N*, *N*-Dimethyltryptamine is typically orally inactive due to a deamination process facilitated by visceral monoamine oxidase enzymes^8^. The technology of the *Ayahuasca* brew orally activates the plant DMT alkaloids by combining them with plants containing beta (β)-carboline alkaloids, a reversible monoamine oxidase inhibitor (MAOI)^3,8^.

Alternate routes of DMT administration, such as inhaled^10–12^, intravenous^13,14^, intramuscular^15^, intra-nasal, and per-rectum reduce the first pass effect resulting in an increase in bioavailability, a more rapid onset, and shorter duration of effect compared to oral administration^10^. Using data from the Global Drug Survey (an anonymous online global survey; n = 22,289), Winstock et al. observed that overall lifetime use of DMT was relatively low (8.9%) and the predominant reported route of administration amongst individuals who used DMT was inhaled (92.2%)^11^.

Early but growing efforts attempt to characterize the phenomenological experience following exogenous DMT administration^2,5,16–30^. Common emerging themes include mystical-type experiences^21^; transcendence and connectedness^2^; increased noesis (ascertaining direct or intuitive knowledge)^31,32^; alterations in consciousness and the sense-of-self^2,17,21^; vivid imagery and perceptual changes^16,24^; emotional effects^16^; death and rebirth^2^; the divine, spirituality, and religiosity^2,21^; and encounters with autonomous entities and beings^5,27^. The early understanding of the DMT experience was largely supported by editorial, non-peer-reviewed, or popular science publications and reports^2,24,25,33–35^. However, recent efforts have been undertaken to substantiate these works with more rigorous systematic methodologies^2,16,17,19,21,27,36^, with few studies specifically examining inhaled-DMT^21,27,37,30^.

In one of the earliest peer-reviewed studies on the subjective effects of DMT, Strassman et al. conducted a unblinded followed by a double-blind randomized controlled trial of intravenous DMT-fumarate in twelve volunteers^16^. A significant dose–response effect of DMT was observed in all six domains of the Hallucinogen Rating Scale (HRS) including: somaesthesia, affect, perception, cognition, volition, and intensity^16,33,38^. To note, the HRS was developed through interviews with individuals experienced with inhaled DMT^16^.

Benny Shanon explored the subjective and perceptual effects of 245 *Ayahuasca* experiences, including 67 of his own experiences^2^. Shanon identified common patterns and attempted to “draw a map” of the *Ayahuasca* experience incorporating themes including, but not limited to, general effects; open-eye visuals; structural typology; the contents and themes of visions; non-visual perceptions; and consciousness^2^. Cott and Rock conducted a survey-based method in 19 DMT users and documented 9 themes that captured the essential aspects of the DMT-induced state following “ingestion”, including hallucinations; entering other realms and contacting other sentient beings; lucidity; affective distortions; ineffability; intensity; spirituality and learning about truths; distortions in sense of time, space, and self; and familiarity^19^.

In a retrospective survey-based study, Griffiths et al. examined the single most memorable “God encounter experience” in individuals who used *N*, *N*-DMT (n = 606) and *Ayahuasca* (n = 435)^21^. A total of 73% and 65% of the *N*, *N*-DMT and *Ayahuasca* experiences, respectively, fulfilled criteria of a complete mystical experience and a large proportion of both cohorts rated the experience as among the most personally meaningful and spiritually significant lifetime experiences^21^.

Davis et al. systematically studied the account of the single most memorable entity encounter after inhaled *N*, *N*-DMT use in 2561 individuals^27^. Almost all of the individuals in this study reported an emotional response to the encounter to which profound and enduring ontological changes in worldview were attributed^27^. Timmermann et al. conducted a fixed-order, placebo controlled, single blind intravenous DMT administration study in 13 participants and established that DMT induced consistent visual, somatic, emotional, and ‘higher-level’ metacognitive effects^14^; and noted similarities between DMT experiences and near-death experiences (NDE)^17^. More recently, Michael et al. performed a thematic analysis from semi-structured interviews of 36 inhaled naturalistic DMT experiences (dose 40–75 mg)^30^. The most prominent theme from this study involved an encounter with “sentient entities that were experienced as beyond”, occurring in 94% of the experiences^30^.

The shared qualities amongst DMT and certain non-drug induced altered states of consciousness^17,18,21,27^, in concurrence with the growing evidence of an endogenous mammalian source of DMT^39,40^, has led to the increasingly accepted hypothesis that endogenous DMT may be responsible for particular alternate states of consciousness^17,21,27^. However, it is not clear that endogenous DMT release occurs in sufficient concentrations or with sufficient selectivity to produce pharmacological effects^41,42^.

There currently exists a paucity of knowledge describing how psychedelics are used in naturalistic settings in Western societies^43^. A greater understanding of the patterns of use of inhaled *N*, *N*-DMT in naturalistic settings and the broader user population will aid in the explication of the expanding research findings^43^. Inhaled *N*, *N*-DMT sometimes occasions profound “ontological shock” experiences prompting a reconsideration of the very nature of reality, and such experiences are associated with enduring positive changes in attitudes, moods and behavior ^5,27^. Given the unusual and consequential nature of these experiences, the current study sought to provide new information about the phenomenology and content of inhaled *N*, *N*-DMT experiences by systematically analyzing thousands of *N*, *N*-DMT experience reports from online postings over a 10-year period. A greater understanding of the inhaled *N*, *N*-DMT experience is critical to further our understanding of the properties and potential of this compound. Such learnings could inform current and future research and therapeutic applications in the management of central nervous system and mental health conditions, and, potentially, catalyze interest in and insights into broader ontological questions pertaining to consciousness and the human psyche^21,27,41,44^.

## Materials and methods

### Data source and collection

All posts to the “DMT” community page (“r/DMT”) on Reddit from database inception in 2009 through to 2018 inclusive (10 years), were screened for inclusion in this study. Reddit is an online social network, news aggregator, and host of forums for communities with common interests to share, discuss, and comment on a topic^45,46^. Data from Reddit has been aggregated previously to inform peer-reviewed research^46–48^. The r/DMT community is a public forum for the discussion and sharing of information pertaining to DMT and is a rich community-based open-access resource, which includes individuals reporting and describing DMT experiences.

Archived posts to the r/DMT community were accessed via Pushshift: a big-data storage project, comprehensive search engine, and real-time analytics tracker for the website Reddit^49^. At the time of this study, Pushshift met the standards for the terms of use of the Reddit application programming interface (API).

**Figure 1:**
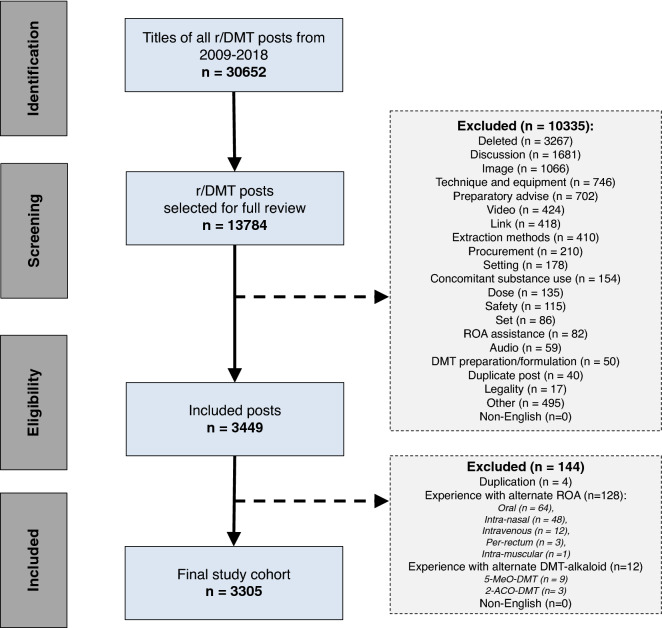
PRISMA flow diagram for screening and selection of r/DMT posts. ROA, route of administration.

The title of all posts to the r/DMT community within the study period were manually screened and included for full post review if the post had the potential to include a self-reported *N*, *N*-DMT experience (see Fig. 1). All relevant posts that were identified after the initial screen underwent full independent manual review to assess candidacy for final study inclusion.

Posts were included within the final study cohort if the post included a firsthand description of any aspect of an inhaled *N*, *N*-DMT experience. Posts were excluded based on the following criteria: (1) the route of administration was not inhaled (i.e., oral, intravenous, intramuscular, rectal, or intra-nasal); (2) the reported DMT alkaloid was not “DMT”, “*N*, *N*-DMT, or “freebase DMT” (i.e., 5-MeO-DMT and 4-ACO DMT were excluded); (3) the post solely referenced or linked to an external website, video, audio file, or image; (4) the post failed to describe any experience or subjective response to *N*, *N*-DMT; (5) the post was non-English; or (6) the post was a duplicate or deleted. Non-English posts were identified but all contained enough information to be excluded based on alternate criteria (i.e., only included a link, image, and/or video). Therefore, zero experiences were excluded for being non-English. All plant-based accompaniments (i.e., Changa, enhanced leaf, and joints or spliffs) were included in this study. Plant-based accompaniments are largely used to improve to vaporization yield and reduce burning the crystalline *N*, *N*-DMT, although it is recognized that Changa also includes MAOI-containing plants. Multiple unique *N*, *N*-DMT experiences reported within a single post were coded as independent and separate experiences.

### Content analysis, statistics, and research ethics

All included posts and experiences underwent independent full manual review. The following data were systematically collected for all posts: month and year of posting, Reddit author username, title, and word count. The following data were systematically collected if available for all experiences: age at time of experience, author gender, previous inhaled *N*, *N*-DMT use, previous psychedelic use, data pertaining to the setting of the experience, plant source used for DMT extraction, the device used for administration, the reported *N*, *N*-DMT dose and number of inhalations, the duration of experience, and concomitant psychoactive substance use.

A grounded theory inductive qualitative analytic coding approach was used for all included experiences using NVivo 12 (12.6.0)^50^. Independent meaningful units (MU) were coded and organized into categorical themes^50,51^. Opportunity for code review, MU development, and thematic development were afforded to four authors (DWL, RCH, CT, and RG) after the completion of the coding of 50, 300, and 1000 experiences. The following thematic categories were developed: (1) physical and somatic experiences; (2) content of visualizations and imagery; (3) entity encounters including entity phenotype, descriptors, attributes, disposition, and characteristics of the interaction; (4) typology, architectural features, structural characteristics, and scenery of the “DMT world”; (5) alerations in consciousness; (6) emotional responses (including positive, rewarding, difficult, and challenging); and (7) statements of profundity.

Additionally, four validated scales for documenting cardinal aspects of the psychedelic experience were completed, including the Mystical Experience Questionnaire (MEQ)^52,53^, Challenging Experience Questionnaire (CEQ)^54^, God Encounter Questionnaire (GEQ)^21^, and Ego-Dissolution Inventory (EDI)^55,56^. The MEQ, CEQ, and EDI were completed for all experiences while the GEQ was completed for all reported entity encounters^27^. To note, the included questionnaires are validated for self-report and not for third-party assessment. However, due to the relative novelty of this field of study and the paucity of validated scales, we felt the application of these questionnaires in this study was still appropriate to facilitate and structure the analysis of the respective features occasioned by DMT. As such, items within the questionnaires were not completed as a rank or scale, but merely as a binary outcome identifying the presence or absence of the questionnaire items within a particular experience.

Statements of profundity were coded and defined as endorsements pronouncing the experience or an aspect of the experience as “the most *[X]* experience of my life” or “…the most *[X]* I have felt in my life…”; where *[X]* was coded as the descriptor. For statements that include multiple descriptors, the order in which the descriptor appeared within a statement was also coded.

### Research ethics board approval

Institutional research ethics board (REB) approval for human research protocols (University of Toronto REB protocol number 39101) was obtained for the collection, analysis, and dissemination of the data and content included within this study.

## Results

A total of 30,652 r/DMT reddit posts were identified and screened over the 10-year period from 2009 to 2018, of which 3305 posts containing 3778 unique inhaled *N*, *N*-DMT experiences were included in this study (see Fig. 1). Two-thousand nine-hundred and thirty-four posts (88.8%) included a report of a single experience, 297 posts (9.0%) included two experiences, and 74 posts (2.2%) included 3 or more experiences.

The included 3305 posts were created by 2277 Reddit authors, with a median age at the time of the *N*, *N*-DMT experience of 23.0 years [interquartile range (IQR), 20.0 to 29.8; reported in 118 experiences (3.1%)], and a median word count of 311 (IQR, 160.0 to 568.0; see Table 1). The gender of the author was identified in 237 experiences (6.3%), including 189 males (5.0%) and 48 females (1.3%). The location in which the experience occurred was reported in 1138 experiences (30.1%), of which the location was most frequently inside (n = 969, 25.6%) within a bedroom or living room (n = 492, 13.0%); while 169 experiences (4.5%) were reported as occurring outside. Music was reportedly used in 408 experiences (10.8%) and the presence or absence of a sitter was identified in 948 experiences (25.1%); of which 157 experiences (4.2%) were completed alone.

**Table 1:** Demographic data of the included posts and *N*, *N*-DMT experiences.

Variables	N (%) or median (IQR)
Posts, n (%)	3305 (100.0)
**Authors, n (%)**	2277 (68.9)
Deleted author, n (%)	351 (10.6)
Experiences, n (%)	3778 (100.0)
Word count, median (IQR)	311.0 (160.0, 568.0)
Age (years), median (IQR)^a^	23.0 (20.0, 29.8)
**Gender**	
Male	189 (5.0)
Female	48 (1.3)
**Setting, n (%)**	
Inside (including inside NOS)	969 (25.6)
Bedroom or living room	492 (13.0)
Vehicle	23 (0.6)
Bathroom, shower, or bath	10 (0.3)
Outside (including outside NOS)	169 (4.5)
Forest, jungle, beach, or tent	39 (1.0)
Backyard, garden, park, or cemetery	22 (0.6)
Music	408 (10.8)
Sitters	
Alone	157 (4.2)
Not alone or sitter present	791 (20.9)
Dose (mg), median (IQR)^b^	40.0 (27.5, 50.0)
Dose (number of inhalations), median (IQR)^c^	3.0 (2.0, 3.0)
**Extraction source**	
*Mimosa hostilis* root bark	95 (2.5)
*Acacia confusa* root bark	34 (0.9)
**Device, n (%)**	
Pipe or glass pipe NOS	598 (15.8)
Vaporizer NOS	253 (6.7)
Bong	185 (4.9)
Dab rig	155 (4.1)
Home-made device ("machine" or bottle-pipe)	138 (3.6)
**Plant-based accompaniments, n (%)**	
Sandwich method	259 (6.8)
Changa	165 (4.4)
Enhanced leaf, joint, or blunt	21 (0.6)
Trip duration (min), median (IQR)^d^	10.0 (5.0, 15.0)
**Previous inhaled DMT use, n (%)**	
0	1008 (26.6)
1	324 (8.6)
Multiple^f^	620 (16.4)
Yes NOS	631 (16.7)
**Previous non-inhaled-DMT psychedelic use, n (%)**^**g**^	
No previous psychedelic use	76 (2.0)
LSD	550 (14.6)
Psilocybin	383 (10.1)
MDMA	80 (2.1)
Other^h^	150 (4.0)
Yes NOS	66 (1.7)
**Concomitant psychoactive substance use, n (%)**^**g**^	536 (14.2)
Cannabis	243 (6.4)
LSD	129 (3.4)
Alcohol or benzodiazepines	101 (2.7)
MDMA	40 (1.1)
Psilocybin	30 (0.8)
Other^i^	33 (0.9)

Reported in ^a^118 experiences (3.1%), ^b^1347 experiences (35.7%), ^c^1087 experiences (28.8%), and ^d^538 experiences (14.2%).

^f^Greater than one previous inhaled DMT experiences or multiple previous experiences NOS.

^g^Does not summate to 100% due to multiple identified within an index experience.

^h^Including Salvia, 2C Class, Ayahuasca, 25B-NBOMe, ketamine, mescaline (or peyote), alpha-methyltryptamine, and ibogaine.

^i^Including ketamine, stimulants, cocaine, opioid, or mescaline.

*4-AcO-DMT* 4-Acetoxy-*N*,*N*-dimethyltryptamine, *5-MeO-DMT* 5-methoxy-*N*,*N*-dimethyltryptamine, *DMT* dimethyltryptamine, *IQR* inter-quartile range, *LSD* lysergic acid diethylamide, *MDMA* 3,4-methyl enedioxy methamphetamine, *mg* milligrams, *min* minutes, *NOS* not otherwise specified.

A median reported *N*, *N*-DMT dose of 40.0 mg (IQR 27.5–50.0) with a median of 3.0 inhalations (IQR 2.0–3.0) per experience were reported in 1347 (35.7%) and 1087 (28.8%) of the experiences, respectively. The plant source of the *N*, *N*-DMT was identified in 129 experiences (3.4%), including 95 (2.5%) experiences involving *N*, *N*-DMT extracted from *Mimosa hostilis* root bark and 34 (0.9%) from *Acacia confusa* root bark. A pipe or glass-pipe (n = 598, 15.8%) was the most frequent reported device used to self-administer the *N*, *N*-DMT. Identified plant-based material accompaniments included the “sandwich method” (containing the DMT between other plants to avoid losing intake due to vaporization produced by directly burning the substance; n = 259, 6.8%); Changa (n = 165, 4.4%); and enhanced-leaf, joints, or blunts (n = 21, 0.6%). The median experience duration, estimated from 538 experiences (14.2%) was 10 min (IQR 5.0–15.0).

No previous inhaled *N*, *N*-DMT experience (i.e., first time-use during the index experience) and one previous inhaled DMT experience were identified in 1008 experiences (26.6%) and 324 experiences (8.6%), respectively (see Table 1). Seventy-six experiences (2.0%) involved the Reddit authors reporting no previous psychedelic use prior to the index experience. Previous experience with lysergic acid diethylamide (LSD) was most frequently reported (n = 550, 14.6%), followed by psilocybin (n = 383, 10.1%), and MDMA (n = 80, 2.1%). Concomitant psychoactive substance use was identified in 536 experiences (14.2%), most frequently involving cannabis (n = 243, 6.4%), followed by LSD (n = 129, 3.4%), alcohol or benzodiazepines (n = 101, 2.7%), MDMA (n = 40, 1.1%), and psilocybin (n = 30, 0.8%). To note, a separate analysis of the data was conducted excluding experiences with reported concomitant psychoactive substance use (n = 3242). No meaningful differences in the distribution of codes or themes were identified in experiences with concomitant psychoactive substance use excluded (see Tables 1, 2, 3, 4, 5, 6, 7, 8,9).

**Table 2:** Physical and somatic responses to *N*, *N*-DMT.

Coded units and themes	Documented in experience, n (%)	
	All experiences (n = 3778)	Experiences with no reported concomitant psychoactive substance use (n = 3242)
**Somatic**		
Somaesthesias	1415 (37.5)	1212 (37.4)
Body vibration, buzz, or tingling	1026 (27.2)	878 (27.1)
Body "high" or euphoria	209 (5.5)	174 (5.4)
Body "load"	180 (4.8)	160 (4.9)
OBE, floating, dissociation, or body dissolving	655 (17.3)	531 (16.4)
Acceleration, falling, or moving at high velocity	332 (8.8)	263 (8.1)
Temperature dysregulation	191 (5.1)	167 (5.2)
Warmth	151 (4.0)	134 (4.1)
Cold	40 (1.1)	33 (1.0)
Unpleasant taste	186 (4.9)	167 (5.2)
Pain (body, abdominal, or oropharyngeal)	69 (1.8)	58 (1.8)
Head pressure, headache, or migraine	53 (1.4)	47 (1.4)
Diaphoresis	31 (0.8)	28 (0.9)
**Auditory-ringing type sound**	583 (15.4)	583 (18.0)
Ringing, buzzing, humming, or vibrating sound	474 (12.5)	406 (12.5)
Static, crackling, "electric", or popping sound	69 (1.8)	58 (1.8)
High pitched tone or tiny tone	40 (1.1)	36 (1.1)
**"Neurologic"**		
Amnestic events	582 (15.4)	582 (17.9)
Partial amnesia	513 (13.6)	513 (15.8)
Full amnesia	69 (1.8)	69 (2.1)
Black out	46 (1.2)	40 (1.2)
Loss of awareness to being under the influence of DMT	46 (1.2)	36 (1.1)
Motor effects	105 (2.8)	92 (2.8)
Paralysis	51 (1.3)	44 (1.4)
Convulsive or athetoid movements, shivering, or shaking	39 (1.0)	34 (1.0)
Motor incoordination or balance impairment	15 (0.4)	14 (0.4)
Dizziness or light headedness	70 (1.9)	60 (1.9)
Synaesthesia	47 (1.2)	42 (1.3)
Facial or oropharyngeal paraesthesias	18 (0.5)	14 (0.4)
**Gastrointestinal**		
Nausea	107 (2.8)	90 (2.8)
Emesis	69 (1.8)	55 (1.7)
Fecal urgency	8 (0.2)	8 (0.2)
**Cardiorespiratory**		
Tachycardia or dysrhythmia	79 (2.1)	68 (2.1)
Cough or lung harshness	70 (1.9)	60 (1.9)
Dyspnea, tachypnea, or apnea	18 (0.5)	17 (0.5)
Chest pressure or discomfort	17 (0.4)	15 (0.5)
Choking sensation	10 (0.3)	9 (0.3)
Breathing difficulty NOS	69 (1.8)	59 (1.8)
**Genitourinary—urinary urgency or incontinence**	24 (0.6)	20 (0.6)

*DMT* dimethyltryptamine, *NOS* not otherwise specified, *OBE* out-of-body experience.

**Table 3:** Content of imagery and visualizations of the *N*, *N*-DMT experiences and changes to visual perception.

Coded units and themes	Documented in experience, n (%)	
	All experiences (n = 3778)	Experiences with no reported concomitant psychoactive substance use (n = 3242)
**Content of imagery and visualizations**		
Fractals, shapes, or patterns	1231 (32.6)	1017 (31.4)
Fractals or geometric shapes	1218 (32.2)	1004 (31)
Kaleidoscope	85 (2.2)	65 (2.0)
Mandala	44 (1.2)	44 (1.4)
Chrysanthemum	32 (0.8)	21 (0.6)
Sacred geometry	22 (0.6)	19 (0.6)
Patterns NOS	325 (8.6)	262 (8.1)
Colours: Vivid, novel, neon, beautiful, hyperintense	953 (25.2)	780 (24.1)
Open eye visuals (OEVs)	499 (13.2)	499 (15.4)
Room and wall distortion (dissolving/melting/warping/breathing)	175 (4.6)	148 (4.6)
Pixilation	39 (1.0)	31 (1.0)
Tracers and halos	20 (0.5)	15 (0.5)
OEVs NOS	463 (12.3)	383 (11.8)
Visual distortion NOS	121 (3.2)	104 (3.2)
Cartoon visuals	113 (3.0)	84 (2.6)
Faces or eyes	90 (2.4)	75 (2.3)
Ancient and/or cultural-based imagery	80 (2.1)	65 (2.0)
"Aztec" or "Mayan" visuals	38 (1.0)	33 (1.0)
Egyptian visuals	33 (0.9)	24 (0.7)
Other^a^	9 (0.2)	8 (0.2)
Visions of past experiences or relatives (dead or alive)	75 (2.0)	60 (1.9)
Writing, symbols, scripture, or hieroglyphs	73 (1.9)	66 (2.0)
Webs or grids	50 (1.3)	41 (1.3)
Visions of previous lives	31 (0.8)	25 (0.8)
Digital or alien visuals	30 (0.8)	27 (0.8)
Flower(s) or lotus	28 (0.7)	25 (0.8)
Tentacles	23 (0.6)	21 (0.6)
Visuals folding-in on themselves	21 (0.6)	18 (0.6)
Animal visuals	17 (0.4)	12 (0.4)
Skulls, skeletons, or other human anatomy	13 (0.3)	10 (0.3)
Satanic visuals	12 (0.3)	12 (0.4)
Closed eye visuals (CEVs) NOS	352 (9.3)	262 (8.1)
**Changes to visual perception**		
Shaky or vibrating vision	139 (3.7)	120 (3.7)
Visual clarity	66 (1.7)	62 (1.9)
Visual field expansion, seeing with eyes closed or with third eye	65 (1.7)	50 (1.5)
Veil or curtain lifting/falling	26 (0.7)	24 (0.7)

^a^Including Islamic, Hindu, Tibetan, North American Indigenous, tribal NOS.

*NOS* not otherwise specified.

**Table 4:** Entity attributes, disposition, and characteristics of the entity interaction.

Coded units and themes	Documented in experience, n (%)	
	All entity encounters (n = 1719)	Entity encounters with no reported concomitant psychoactive substance use (n = 1453)
**Positive**^**a**^	600 (34.9)	600 (41.3)
Benevolent (i.e. kind, compassionate, altruistic)	527 (30.7)	527 (36.3)
Comforting, protecting, or outwardly caring	230 (13.4)	168 (11.6)
Welcoming	177 (10.3)	174 (12.0)
Loving or embracing	139 (8.1)	120 (8.3)
Dancing, singing, partying	122 (7.1)	104 (7.2)
Healing	51 (3.0)	48 (3.3)
Sexual, intimate, or sensual	29 (1.7)	24 (1.7)
Happy, friendly, or excited	26 (1.5)	20 (1.4)
Provision of nourishment	13 (0.8)	12 (0.8)
Affusion or aspersion-type action or pouring liquid on individual	10 (0.6)	10 (0.7)
**Companion-, pedagogical-, or guide-type interaction**	557 (32.4)	544 (37.4)
Guiding, touring, or showing	457 (26.6)	337 (23.2)
Testing or offering a choice (including option to live or die)	77 (4.5)	64 (4.4)
Controlling or altered the visuals of the experience	58 (3.4)	46 (3.2)
Transferred knowledge	52 (3.0)	45 (3.1)
Beckoning or summoning	47 (2.7)	41 (2.8)
Encouraging	24 (1.4)	23 (1.6)
Offered gifts or information	18 (1.0)	15 (1.0)
**Negative or difficult**	196 (11.4)	196 (13.5)
Menacing, malicious, evil, threatening, violent, attacking, intimidating, or bullying	92 (5.4)	75 (5.2)
Angry, unfriendly, unhappy, disappointed, frustrated, or sad	57 (3.3)	42 (2.9)
Rejecting, denying, or unwelcoming	45 (2.6)	36 (2.5)
Torturing or raping	14 (0.8)	12 (0.8)
Teared apart or eaten/consumed by entity	11 (0.6)	9 (0.6)
**Medical-type interaction**	154 (9.0)	154 (10.6)
Examining, observing, scanning, or analyzing	141 (8.2)	111 (7.6)
Implantation of device	59 (3.4)	51 (3.5)
Surgery, procedure, operation, injection, or experimentation	56 (3.3)	53 (3.6)
Exploration or probing	34 (2.0)	29 (2.0)
**Neutral or other**	181 (10.5)	181 (12.5)
Playful or mischievous	77 (4.5)	68 (4.7)
Forehead touched or manipulated	16 (0.9)	13 (0.9)
Chest touched or manipulated	11 (0.6)	10 (0.7)
**Language and communication**	163 (9.5)	141 (9.7)
Novel or alien language	152 (8.8)	131 (9.0)
Communication via emotions, colours, or vibrations	11 (0.6)	10 (0.7)

^a^In addition to entity attributes identified in the God Encounter Questionnaire (see Table 5).

**Table 5:** God Encounter Questionnaire (GEQ).

Questionnaire items	Documented in experience, n (%)	
	All entity encounters (n = 1719)	Entity encounters with no reported concomitant psychoactive substance use (n = 1453)
**Details of encounter**		
Went into the experience with the intention of encountering that which was encountered	38 (2.2)	32 (2.2)
The encounter was initiated by that which was encountered (not by [the author])	1681 (97.8)	1421 (97.8)
**Senses with which you interacted during the encounter**		
Visual	1520 (88.4)	1289 (88.7)
Auditory	475 (27.6)	413 (28.4)
Tactile/bodily sensation	257 (15.0)	222 (15.3)
Taste or smell	3 (0.2)	3 (0.2)
Extra-sensory	429 (25.0)	361 (24.8)
**Communication**		
There was communication (1-way or 2-way exchange of information)	1279 (74.4)	1099 (75.6)
Communication was a 2-way exchange of information	442 (25.7)	375 (25.8)
Communication was a 1-way exchange of information (from the entity to the author)	826 (48.0)	714 (49.1)
Communication was a 1-way exchange of information (from the author to the entity)	11 (0.6)	13 (0.9)
**Communication type**		
Visual (e.g. gestures)	937 (54.5)	801 (55.1)
Verbal-auditory	489 (28.4)	427 (29.4)
Somatic (e.g. touch/kinesthetic)	250 (14.5)	215 (14.8)
Extrasensory-telepathic	361 (21.0)	303 (20.9)
**Immediate results of the encounter**		
Had an emotional response during the encounter	1583 (92.1)	1349 (92.8)
That which was encountered had an emotional response during the encounter	394 (22.9)	326 (22.4)
Ascertained a message, task, mission, or insight from the encounter	792 (46.1)	673 (46.3)
Acquired predictions about the future	6 (0.3)	4 (0.3)
**Attributes to that which was encountered**		
Benevolent	527 (30.7)	441 (30.4)
Intelligent	1238 (72.0)	1066 (73.4)
Sacred	238 (13.8)	181 (12.5)
Conscious (i.e. self-aware)	1602 (93.2)	1364 (93.9)
Eternal	175 (10.2)	140 (9.6)
All-knowing	320 (18.6)	266 (18.3)
Agency (e.g. could it affect outcomes, events, or material objects in this reality)	232 (13.5)	197 (13.6)
Petitionable (e.g. in response to prayer or petition, it might change events or circumstances)	452 (26.3)	384 (26.4)
Positively judgmental (e.g. inclined toward strong approval or reward)	800 (46.5)	680 (46.8)
Negatively judgmental (e.g. inclined toward strong disapproval or harsh punishment)	308 (17.9)	256 (17.6)
**Additional interpretation of that which was encountered**		
That which was encountered existed, as least in part, in some other dimension or reality	1504 (87.5)	1282 (88.2)
Was completely the same as that which was encountered	48 (2.8)	33 (2.3)
That which was encountered continued to exist after the encounter	1117 (65.0)	944 (65.0)

**Table 6:** Typology, architectural features, structural characteristics, scenery, and objects encountered in the "DMT world".

Coded units and themes	Documented in experience, n (%)	
	All experiences (n = 3778)	Experiences with no reported concomitant psychoactive substance use (n = 3242)
**Alternate or higher dimensions**	952 (25.2)	154 (4.8)
Hyperspace	646 (17.1)	624 (19.2)
1- or 2-dimensional experience	30 (0.8)	24 (0.7)
4- or 5-dimensional experience	56 (1.5)	47 (1.4)
Alternate, hyper-, or multi-dimensional NOS	220 (5.8)	83 (2.6)
**Room(s)**	582 (15.4)	574 (17.7)
Waiting room	105 (2.8)	93 (2.9)
Sterile, clean and/or functional room	88 (2.3)	77 (2.4)
Medical room, operating room, examination room, or hospital	60 (1.6)	53 (1.6)
Office space, classroom, or school	13 (0.3)	12 (0.4)
Laboratory or control room	6 (0.2)	5 (0.2)
Nursery, crib, or spa	5 (0.1)	3 (0.1)
Kitchen or cafeteria	4 (0.1)	4 (0.1)
Geometric, fractal, or multi-coloured room	79 (2.1)	66 (2.0)
White or bright room	39 (1.0)	32 (1.0)
Dark, black, or black-and-white room	20 (0.5)	16 (0.5)
Pink, golden, or blue room	19 (0.5)	17 (0.5)
Room or large room NOS	232 (6.1)	196 (6.0)
**Tunnel or tunnel-type structure**	390 (10.3)	341 (10.5)
**Void (white, golden, black, or NOS)**	233 (6.2)	188 (5.8)
**Landscape and landscape features**	229 (6.1)	170 (5.2)
Alien, fractal, or golden landscape	46 (1.2)	30 (0.9)
Urban landscape, cities, towns	39 (1.0)	27 (0.8)
Fields	26 (0.7)	21 (0.6)
Jungle, forest, or ancient forest	22 (0.6)	18 (0.6)
Beach, ocean, lake, or island	16 (0.4)	13 (0.4)
Desert or savannah	12 (0.3)	7 (0.2)
Tree(s) or tree of life	12 (0.3)	12 (0.4)
Garden, fountain, waterfall, or pool	10 (0.3)	8 (0.2)
Mountains	9 (0.2)	7 (0.2)
Landscape NOS	37 (1.0)	27 (0.8)
**Astronomical themes (stars, planets, celestial bodies, or galaxies, or outer space NOS)**	212 (5.6)	170 (5.2)
**White, bright, or beam/stream of light**	130 (3.4)	110 (3.4)
**Machinery, clockwork, gears, or wheels**	78 (2.1)	61 (1.9)
**Ancient or cultural-specific architectural themes**	70 (1.9)	58 (1.8)
Egyptian themed typology	33 (0.9)	22 (0.7)
Indigenous Meso-/South American structures	30 (0.8)	30 (0.9)
Ancient Greco-Roman or Norse typology	7 (0.2)	6 (0.2)
**Carnival, circus, or playhouse**	53 (1.4)	44 (1.4)
**Vehicles**	49 (1.3)	44 (1.4)
Spaceship	37 (1.0)	33 (1.0)
Train, roller coaster, or vehicle NOS	12 (0.3)	11 (0.3)
**Pyramid(s)**	44 (1.2)	35 (1.1)
**Place of Worship (temple, altar, cathedral, mosque, or monastery)**	31 (0.8)	28 (0.9)
**Hell**	31 (0.8)	23 (0.7)
**Domed or large spherical area**	22 (0.6)	19 (0.6)
**Heaven or Nirvana**	21 (0.6)	14 (0.4)
**Alien-, future-, or advanced-technology**	18 (0.5)	18 (0.6)
**Other buildings or structures**^**a**^	111 (2.9)	100 (3.1)
**Objects**	352 (9.3)	289 (8.9)
Spherical objects, globes, or orbs	46 (1.2)	37 (1.1)
Cubes or containers (colourful, Rubik's cubes, fractal cubes)	30 (0.8)	29 (0.9)
Helices, DNA, spirals, or hourglass shapes	22 (0.6)	17 (0.5)
Impossible objects, tesseract, or 4-dimensional objects	16 (0.4)	10 (0.3)
Ribbons or toys	13 (0.3)	11 (0.3)
Object(s) NOS	225 (6.0)	185 (5.7)

^a^Including castle, palace, house, apartment, cabin, cave, underground space, factory, garage, train yard, bar, pub, mall, shop, store, zoo, casino, airport, jail, dungeon, library, or museum. *DNA* deoxyribonucleic acid, *NOS* not otherwise specified.

**Table 7:** Rewarding emotional responses and the Mystical Experience Questionnaire (MEQ).

Coded units, themes, and questionnaire items	Documented in experience, n (%)	
	All experiences (n = 3778)	Experiences with no reported concomitant psychoactive substance use (n = 3242)
**Rewarding responses**		
Familiarity	482 (12.8)	423 (13.0)
Familiar	428 (11.3)	369 (11.4)
Feels like "home" or sense of belonging	55 (1.5)	47 (1.4)
Tearful and tears of joy	111 (2.9)	90 (2.8)
Euphoria	93 (2.5)	79 (2.4)
Accepting death or removed fear of dying	59 (1.6)	48 (1.5)
Rebirth	53 (1.4)	40 (1.2)
Sexual or intimate energy or experience	33 (0.9)	33 (1.0)
Feels real or more real than everyday reality	22 (0.6)	22 (0.7)
**Mystical Experience Questionnaire (MEQ)**		
Mystical	1188 (31.4)	980 (30.2)
Freedom from the limitations of your personal self and feeling a unity or bond with what was felt to be greater than personal self	331 (8.8)	270 (8.3)
Experience of pure being and pure awareness (beyond the world of sense impressions)	133 (3.5)	105 (3.2)
Experience of oneness in relation to an “inner world” within	141 (3.7)	111 (3.4)
Experience of the fusion of your personal self into a larger whole	184 (4.9)	147 (4.5)
Experience of unity with ultimate reality	258 (6.8)	211 (6.5)
Feeling that [the author] experienced eternity or infinity	266 (7.0)	212 (6.5)
Experience of oneness or unity with objects and/or persons perceived in your surroundings	159 (4.2)	125 (3.9)
Experience of the insight that “all is One”	116 (3.1)	92 (2.8)
Awareness of the life or living presence in all things	93 (2.5)	77 (2.4)
Gain of insightful knowledge experienced at an intuitive level	717 (19.0)	593 (18.3)
Certainty of encounter with ultimate reality (in the sense of being able to “know” and “see” what is really real at some point during your experience	335 (8.9)	270 (8.3)
Convinced now that [the author] encountered ultimate reality (i.e., that you “knew” and “saw” what was really real)	328 (8.7)	267 (8.2)
Sense of being at a spiritual height	295 (7.8)	254 (7.8)
Sense of reverence	924 (24.5)	755 (23.3)
Feeling that [the author] experienced something profoundly sacred and holy	204 (5.4)	168 (5.2)
Positive mood	2984 (79.0)	2524 (77.9)
Experience of amazement	2715 (71.9)	2297 (70.9)
Feelings of tenderness and gentleness	1078 (28.5)	910 (28.1)
Feelings of peace and tranquility	1058 (28.0)	892 (27.5)
Experience of ecstasy	239 (6.3)	186 (5.7)
Sense of awe or awesomeness	2671 (70.7)	2253 (69.5)
Feelings of joy	2136 (56.5)	1805 (55.7)
Transcendence of space or time	2746 (72.7)	2320 (71.6)
Loss of usual sense of time	362 (9.6)	287 (8.9)
Loss of usual sense of space	2710 (71.7)	2289 (70.6)
Loss of usual awareness of where you were	1884 (49.9)	1577 (48.6)
Sense of being “outside of” time, beyond past and future	174 (4.6)	137 (4.2)
Being in a realm with no space boundaries	828 (21.9)	665 (20.5)
Experience of timelessness	151 (4.0)	116 (3.6)
Ineffability	543 (14.4)	448 (13.8)
Sense that the experience cannot be described adequately in words	534 (14.1)	439 (13.5)
Feeling that [the author] could not do justice to experience by describing it in words	512 (13.6)	423 (13.0)
Feeling that it would be difficult to communicate [author’s] own experience to others who have not had similar experiences	80 (2.1)	65 (2.0)

**Table 8:** Ego-Dissolution Inventory (EDI).

Questionnaire items	Documented in experience, n (%)	
	All experiences (n = 3778)	Experiences with no reported concomitant psychoactive substance use (n = 3242)
Felt especially assertive	4 (0.1)	4 (0.1)
Experienced a dissolution of “self” or ego	140 (3.7)	129 (4.0)
Felt more important or special than others	4 (0.1)	3 (0.1)
Felt at one with the universe	112 (3.0)	104 (3.2)
Ego felt inflated	4 (0.1)	4 (0.1)
Felt a sense of union with others	82 (2.2)	76 (2.3)
Felt especially sure-of-myself	6 (0.2)	6 (0.2)
Experienced a decrease in sense of self-importance	92 (2.4)	83 (2.6)
Felt especially keen and competitive	4 (0.1)	3 (0.1)
Experienced a disintegration of “self” or ego	140 (3.7)	124 (3.8)
Felt like viewpoint was worth more than other peoples’	3 (0.1)	3 (0.1)
Felt far less absorbed by own issues and concerns	81 (2.1)	74 (2.3)
Felt especially self-confident	6 (0.2)	6 (0.2)
Lost all sense of ego	134 (3.5)	122 (3.8)
Felt especially self-assured	6 (0.2)	6 (0.2)
All notion of self and identity dissolved away	134 (3.5)	124 (3.8)

**Table 9:** Difficult emotional responses and the non-physical components of the Challenging Experience Questionnaire (CEQ).

Coded units, themes, and questionnaire items	Documented in experience, n (%)	
	All experiences (n = 3778)	Experiences with no reported concomitant psychoactive substance use (n = 3242)
**Difficult emotional responses**		
Time loop	31 (0.8)	22 (0.7)
Claustrophobia, feeling trapped, or feeling lost	17 (0.4)	15 (0.5)
Thoughts or mind racing	9 (0.2)	9 (0.3)
Suicidality	9 (0.2)	6 (0.2)
**Challenging Experience Questionnaire (CEQ)**		
Fear	840 (22.2)	700 (21.6)
Frightened	680 (18.0)	570 (17.6)
Panic	252 (6.7)	211 (6.5)
Experience of fear	693 (18.3)	580 (17.9)
Anxious	468 (12.4)	383 (11.8)
Had the feeling something horrible would happen	402 (10.6)	335 (10.3)
Grief	416 (11.0)	350 (10.8)
Sad	60 (1.6)	45 (1.4)
Feelings of grief	65 (1.7)	51 (1.6)
Despair	140 (3.7)	117 (3.6)
Feel like crying	42 (1.1)	30 (0.9)
Feelings of despair	137 (3.6)	114 (3.5)
Emotional and/or physical suffering	386 (10.2)	326 (10.1)
Insanity	107 (2.8)	87 (2.7)
Fear that [the author] might lose [their] mind or go insane	69 (1.8)	56 (1.7)
Change in sense of sanity	80 (2.1)	66 (2)
Was afraid that the state would last forever	92 (2.4)	74 (2.3)
Isolation	45 (1.2)	35 (1.1)
Felt isolated from everything and everyone	43 (1.1)	33 (1.0)
Feel isolated from people and things	44 (1.2)	34 (1.0)
Experience of Isolation and loneliness	44 (1.2)	34 (1.0)
Death	236 (6.2)	188 (5.8)
Profound experience of own death	160 (4.2)	128 (3.9)
Feel as if dead or dying	227 (6.0)	178 (5.5)
Paranoia	17 (0.4)	15 (0.5)
Feeling that people were plotting against [the author]	9 (0.2)	7 (0.2)
Experience of antagonism toward people around [the author]	11 (0.3)	10 (0.3)

### Physical and somatic experiences

Somaesthesias (i.e., bodily sensations or kinesthetic hallucinations) were identified in 1415 experiences (37.5%) including a body vibration, buzz, or tingling (n = 1026, 27.2%); body “high” or body euphoria (n = 209, 5.5%); and body “load” (n = 180, 4.8%; see Table 2).

An out of body experience (OBE; including floating out of body, body dissolving, spirit/soul leaving body, falling away from body) was reported in 655 experiences (17.3%). A sensation of accelerating, falling, or moving at a high velocity was identified in 332 experiences (8.8%).

Temperature dysregulation was identified in 191 experiences (5.1%) with a sensation of warmth (n = 151, 4.0%) more frequently endorsed than cold (n = 40, 1.1%). An unpleasant taste was reported in 186 experiences (4.9%). Pain (body, abdominal, or oropharyngeal) was endorsed in 69 experiences (1.8%); head pressure, headache, or migraines in 53 experiences (1.4%); and diaphoresis in 31 experiences (0.8%).

An auditory ringing-type sound was reported in 583 experiences (15.4%). Descriptors for this sound included ringing, buzzing, vibrating, humming, static, crackling, "electric", popping, a high pitched or tinny tone, droning, pulsing, hissing, whining noise, and an auditory carrier wave.

Amnestic events were identified in in 582 experiences (15.4%). Partial amnesia of the experience was reported in 513 experiences (13.6%) while full amnesia and “blackouts” were reported in 69 (1.8%) and 46 (1.2%) experiences, respectively. A loss of awareness to being under the influence of *N*, *N*-DMT during the actual experience was identified in 46 experiences (1.2%).

Motor effects were identified in 105 experiences (2.8%), most commonly a sense of paralysis (n = 51, 1.3%), followed by convulsive- or athetoid-type movements (n = 39, 1.0%) and motor incoordination or balance impairment (n = 15, 0.4%).

Dizziness or light headedness were endorsed in 70 experiences (1.9%). Synaesthesia (including audiovisual synaesthesia) was identified in 47 experiences (1.2%). Facial or oropharyngeal paraesthesias were endorsed in 18 experiences (0.5%).

Predominant gastrointestinal complaints included nausea (n = 107, 2.8%) and emesis (n = 69, 1.8%). A sense of fecal urgency was reported in 8 experiences (0.2%), while urinary urgency or incontinence was reported in 24 experiences (0.6%).

A sense of tachycardia or dysrhythmia was reported in 79 experiences (2.1%), while a cough or lung harshness; dyspnea, tachypnea, or apnea; chest pressure; choking sensation; and breathing difficulty not otherwise specified (NOS) were reported in 70 (1.9%), 18 (0.5%), 17 (0.4%), 10 (0.3%), and 69 (1.8%) of experiences, respectively.

### Content of visualizations and imagery

The most frequently reported visualizations included fractals, geometric shapes, and patterns [n = 1231, 32.6%; including kaleidoscopes (n = 85, 2.2%), mandalas (n = 44, 1.2%), chrysanthemums (n = 32, 0.8%), and sacred geometry (n = 22, 0.6%)] and vivid, novel, neon, beautiful, or hyperintense colours (n = 953, 25.2%; see Table 3).

Four-hundred and ninety-nine experiences (13.2%) described open eye visuals (OEVs) including room and wall distortion (n = 175, 4.6%), pixilation (n = 39, 1.0%), and tracers or halos (n = 20, 0.5%). Cartoon-type visuals were reported in 113 experiences (3.0%) and the description of faces or eyes were identified in 90 experiences (2.4%).

Ancient and/or cultural-associated imagery (i.e., ancient Egyptian or indigenous Meso-/South American) was reported in 80 experiences (2.1%). Visions of relatives (dead or alive) and visions of “previous lives” were described in 75 (2.0%) and 31 (0.8%) experiences, respectively. Additional content of visualizations and imagery are listed in Table 3.

### Changes to visual perception

Changes in visual perception included a shaky or vibrating vision (n = 139, 3.7%; see Table 3); visual clarity (n = 66, 1.7%); visual field expansion, seeing with eyes closed or with a third eye (n = 65, 1.7%); and a perceptual veil or curtain lifting/falling (n = 26, 0.7%).

### Entity encounters and entity phenotype

**Figure 2:**
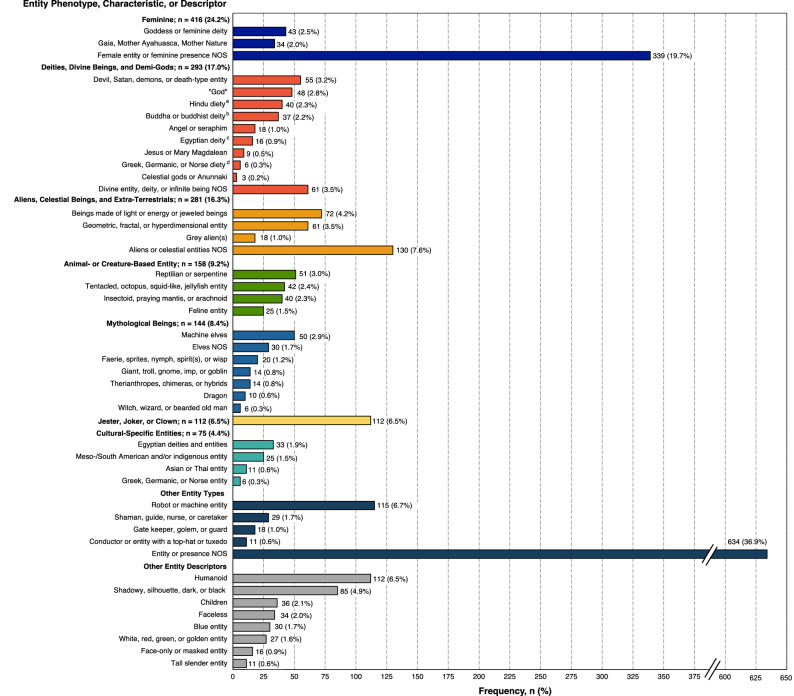
Frequency and distribution of reported entity phenotypes, characteristics, and attributes. Prevalence rates reflect the frequency in relation to experiences that reported an entity encounter (n = 1719) and do not summate to 100% due to instances whereby multiple entity phenotypes, characteristics, and/or descriptors were included within a single experience and/or were used to describe a particular entity. ^a^Including Ganesh, Shiva, Kali, Indra, Naga, or Shakti; ^b^including Tara; ^c^including Ra, Anubis, and pharaohs; ^d^including Minerva, Zeus, and Thor.

One of the most salient themes of the *N, N-*DMT experiences involved encounters and interactions with seemingly autonomous entities, occurring in 45.5% (n = 1719) experiences. Encounters with an archetypal feminine entity were most frequent (n = 416, 24.2% of entity encounters; see Fig. 2) and included a goddess or feminine deity (n = 43, 2.5%); Gaia, Mother Ayahuasca, or Mother Nature (n = 34, 2.0%); and a female entity or feminine presence not otherwise specified (NOS; n = 339, 19.7%).

The next most frequent entity phenotype was that of deities, divine beings, and demi-gods encountered in 293 experiences (17.0%); most frequently identified were the Devil, Satan, demons, or death-type entities (n = 55, 3.2%) followed by “God” (n = 48, 2.8%). Hindu deities, Buddhist (including Buddha) deities, and ancient Egyptian deities were encountered in 40 (2.3%), 37 (2.2%), and 16 (0.9%) experiences, respectively. Angels or seraphim were encountered in 18 experiences (1.0%), while Jesus or Mary Magdalene were identified in 9 experiences (0.5%). See Fig. 2 for additional encountered deity-type entities.

Aliens, celestial beings, and extra-terrestrials were encountered in 281experiences (16.3%). Included within this thematic phenotype were beings made of light or energy and jeweled beings (n = 74, 4.2%); geometric, fractal, or hyperdimensional entities (n = 61; 3.5%); grey aliens (n = 18, 1.0%); and aliens or celestial beings NOS (n = 130; 7.6%).

Animal-based and creature-based entities were reported in 158 of the entity experiences (9.2%), encompassing reptilian or serpentine entities (n = 51, 3.0%); tentacled entities, octopus-like, squid-like, and jellyfish-like entities (n = 42, 2.4%); insectoid, praying mantis, or arachnoid entities (n = 40, 2.3%); and feline entities (n = 25, 1.5%).

Mythological beings were described in 144 experiences (8.4%). Machine elves were encountered in 50 experiences (2.9%); elves NOS in 30 (1.7%); faerie, sprites, nymph, spirits, or wisps in 20 (1.2%); giants, trolls, gnomes, imps, or goblins in 14 (0.8%); therianthropes, chimeras, and hybrids in 14 (0.8%); dragons in 10 (0.6%); and witches, wizards, or a bearded individual in 6 (0.3%).

A jester, joker, or clown was described in 112 encounters (6.5%).

Cultural-specific entities were encountered in 75 experiences (4.4%); most commonly ancient Egyptian deities and entities (n = 33, 1.9%); indigenous Meso-/South American entities (n = 25, 1.5%); Asian or Thai entities (n = 11, 0.6%); and Greek, Germanic, or Norse entities (n = 6, 0.3%).

Other phenotypes included a robot or machine entity (n = 115, 6.7%); a shaman, guide, nurse, or caretaker (n = 29, 1.7%); a gate keeper, golem, or guard (n = 18, 1.0%); and a conductor or entity with a top-hat or tuxedo (n = 11, 0.6%).

Other entity descriptors included humanoid (n = 112, 6.5%); shadowy, silhouette, dark, or black entities (n = 85, 4.9%); children (n = 36, 2.1%); faceless (n = 34, 2.0%); blue entity (n = 30, 1.7%); white, red, green, or golden entity (n = 27, 1.6%); face-only or masked entity (n = 16, 0.9%); and tall slender entity (n = 11, 0.6%).

### Entity attributes, disposition, and characteristics of the interaction

The attributes, disposition, and characteristics of the entities and the encounters were identified and coded (see Table 4). Positive interactions occurred in 600 encounters (34.9%) and most frequently involved a benevolent (i.e., kind, compassionate, altruistic, etc.) interaction (n = 527; 30.7%); followed by an interaction characterized as comforting, protecting, or outwardly caring in 230 encounters (13.4%); welcoming (n = 177, 10.3%); loving or embracing (n = 139, 8.1%); and dancing, singing, or partying (n = 122, 7.1%).

The Reddit author reported being healed by an entity in 51 entity encounters (3.0%). A sexual, intimate, or erotic interaction was documented in 29 experiences (1.7%) and a happy, friendly, or excited interaction in 26 experiences (1.5%). An interaction involving the Reddit author being provided nourishment occurred in 13 experiences (0.8%). An affusion-type or aspersion-type action or the entity pouring liquid on the Reddit author was described in 10 experiences (0.6%).

A companion-type, pedagogical, or guide-type interaction was identified in 557 encounters (32.4%). An entity guiding, touring, or showing occurred in 457 experiences (26.6%), while an entity beckoning or summoning occurred in 47 experiences (2.7%). The entity tested or offered a choice (including option to live or die) to the Reddit author in 77 experiences (4.5%). An entity controlled or altered the visuals of the experience in 58 encounters (3.4%), transferred knowledge in 52 encounters (3.0%), and was encouraging in 24 (1.4%). Gifts or information were offered by entities in 18 experiences (1.0%).

A negative or difficult entity interaction was described in 196 of the 1719 entity encounters (11.4%), most frequently involving a description of an entity being menacing, maliciousness, evil, threatening, violent, attacking, intimidating, or bullying in 92 experiences (5.4%). An angry, frustrated, unfriendly, unhappy, disappointed, or sad entity disposition was reported in 57 encounters (3.3%). A rejecting, denying, or unwelcoming encounter was reported in 45 experiences (2.6%). A description of being tortured or raped by an entity occurred in 14 experiences (0.8%), while being teared apart or eaten/consumed by an entity occurred in 11 encounters (0.6%).

A medical-type interaction was coded in 154 encounters (9.0%), most frequently involving the description of an entity examining, observing, scanning, or analyzing the Reddit author (n = 141, 8.2%). The implantation of a device was described in 59 encounters (3.4%); a surgery, procedure, operation, injection, or experimentation in 56 encounters (3.3%); and bodily exploration or probing in 34 encounters (2.0%).

An entity was playful or mischievous in 77 experiences (4.5%). An entity touched or interacted with the Reddit author's forehead or chest in 16 (0.9%) and 11 (0.6%) experiences, respectively.

The description of a novel or alien language was described in 152 experiences (8.8%), while communication via emotions, colours, or vibrations was reported 11 experiences (0.6%).

### God Encounter Questionnaire (GEQ)

In the majority of entity encounters, the encounter was initiated by the entity (n = 1681, 97.8%; see Table 5) and sensed visually (n = 1520, 88.4%), followed by auditorily (n = 475, 27.6%), extra-sensorially (n = 429, 25.0%), by tactile/bodily sensation (n = 257, 15.0%), and taste or smell (n = 3, 0.2%).

Entity communication occurred in 1279 of the 1719 entity experiences (74.4%) which was most frequently one-way from the entity to the Reddit author (n = 826, 48.0%), followed by two-way (n = 442, 25.7%), and one-way from the Reddit author to the entity (n = 11, 0.6%). Communication was most frequently visual (n = 937, 54.5%), followed by verbal-auditory (n = 489, 28.4%), extra-sensory or telepathic (n = 361, 21.0%), and somatic (n = 250, 14.5%).

The majority of the Reddit authors had an emotional response during the encounter (n = 1583, 92.1%) and that which was encountered had an emotional response during the encounter in 394 experiences (22.9%). The Reddit author ascertained a message, task, mission, or insight from the encounter in 792 experiences (46.1%) and very few acquired predictions about the future (n = 6, 0.3%).

Additional attributes of the entity included conscious (i.e., self-aware; n = 1602, 93.2%); intelligent (n = 1238, 72.0%); benevolent (n = 527, 30.7%); petitionable (n = 452, 26.3%); all-knowing (n = 320, 18.6%), sacred (n = 238, 13.8%), agency (n = 232, 13.5%), and eternal (n = 175, 10.2%). A positively judgmental entity interaction occurred in 800 experiences (46.5%) while a negatively judgmental interaction occurred in 308 experiences (17.9%).

That which was encountered existed, at least in part, in some other dimension or reality in 1504 experiences (87.5%) and continued to exist after the encounter in 1117 experiences (65.0%). The Reddit author felt they were completely the same as that which was encountered in 48 experiences (2.8%).

### Typology, architectural features, structural characteristics, scenery, and objects of the “DMT world”

Common typological, structural, architectural features were identified and catalogued (see Table 6). Descriptions of alternate or higher dimensions were most frequently identified occurring in 952 experiences (25.2%) and included an experience of 1- or 2-dimensions (n = 30, 0.8%); 4- or 5-dimensions (n = 56, 1.5%); hyperspace (n = 646, 17.1%); and alternate-, hyper-, or multi-dimensions NOS (n = 220, 5.8%). To note, it is recognized that an interaction with an entity that existed, at least in part, in some other dimension or reality occurred in 1504 experiences (39.8%); however, authors frequently reported interacting with entities from a different dimension/reality whilst not being present in that dimension/reality themselves.

The description of being inside a room was identified in 582 experiences (15.4%); including 105 (2.8%) that identified being in the “waiting room,” specifically.

Reports of a sterile, clean, or functional room (including a medical, operating, or examination rooms) occurred in 88 experiences (2.3%). A geometric, fractal, or multi-coloured room was reported in 79 experiences (2.1%), while a white or bright room was described in 39 experiences (1.0%). See Table 6 for additional descriptions of rooms encountered.

A tunnel-type structure was reported in 390 experiences (10.3%). A void (including a white, golden, black, and void NOS) was described in 233 experiences (6.2%).

Landscape and landscape features were described in 229 experiences (6.1%), most frequently, an alien, fractal, or golden landscape (n = 46, 1.2%); an urban landscape (n = 39, 1.0%); fields (n = 26, 0.7%); a jungle, forest, or ancient forest (n = 22, 0.6%); and a beach, ocean, lake, or island (n = 16, 0.4%). See Table 6 for additional landscape descriptors.

Astronomical themes (including stars, planets, celestial bodies, galaxies, and outer space NOS) were encountered in 212 experiences (5.6%). A white, bright, or beam/stream of light was described in 130 experiences (3.4%) and machinery, clockwork, gears, or wheels were reported in 78 experiences (2.1%).

Ancient and/or cultural-specific architectural themes were encountered in 70 experiences (1.9%), including ancient Egyptian typology in 33 (0.9%), indigenous Meso-/South American (i.e., Aztecan, Mayan, etc.) in 30 (0.8%), and ancient Greco-Roman or Norse in 7 (0.2%).

A carnival, circus, or playhouse was encountered in 53 experiences (1.4%). Forty-nine experiences (1.3%) involved a vehicle, most commonly a spaceship (n = 37, 1.0%).

Pyramids were encountered in 44 experiences (1.2%) and a place of worship was identified in 31 experiences (0.8%). Metaphysical hell and heaven/nirvana were described in 31 (0.8%) and 21 (0.6%) experiences, respectively. Reddit authors reported being inside a domed or large sphere in 22 occurrences (0.6%). Eighteen (0.5%) Reddit authors reported encountering alien, advanced, or future technology.

Objects were frequently encountered (n = 352, 9.3%), with the most common being spherical objects [including globes and orbs (n = 46, 1.2%); cubes or containers (n = 30, 0.8%); and helices, deoxyribonucleic acid (DNA), spirals, or hourglass-shapes (n = 22, 0.6%)].

### Rewarding emotional responses

A sense of familiarity to the experience was identified in 482 experiences (12.8%), of which 55 experiences (1.5%) reporting a sense of “home” or belonging (see Table 7).

Becoming tearful or reporting tears of joy were reported in 111 experiences (2.9%) and euphoria was reported in 93 experiences (2.5%). Despite 236 reports (6.2%) indicating a “feeling as if dead or dying” or a “profound experience of death” (see Table 9), 59 experiences (1.6% of all experiences or 25.0% experiences including the theme of death) endorsed an acceptance of death or the removal of fear of death.

Fifty-three experiences (1.4%) described rebirth; 33 experiences (0.9%) incorporated a sexual, intimate, or erotic tone; and 22 reports (0.6%) stated the experience felt more real than everyday reality.

### Mystical Experience Questionnaire (MEQ)

A total 2696 experiences (71.4%) reported a minimum of one item on the MEQ, including 1188 experiences (31.4%, see Table 7) containing at least one item within the “mystical” subsection of the MEQ, most frequently a “gain of insightful knowledge experienced at an intuitive level” (n = 717, 19.0%). A total of 2984 experiences (79.0%) included reports coded within at least one item within the “positive mood” subsection of the MEQ, most frequently an “experience of amazement” (n = 2715, 71.9%). A total of 2746 experiences (72.7%) included at least one item within the “transcendence of space or time” subsection of the MEQ, most frequently a “loss of your usual sense of space” (n = 2710, 71.7%). A total of 543 experiences (14.4%) reported at least one item within the “ineffability” subsection of the MEQ, most frequently a “sense that the experience cannot be described adequately in words” (n = 534, 14.1%).

### Ego-Dissolution Inventory (EDI)

A total of 259 experiences (6.9%) endorsed at least one item on the EDI, most frequently experience of a ‘dissolution of “self” or ego’ (n = 140, 3.7%; see Table 8) and experience of a disintegration of “self” or ego’ (n = 140, 3.7%). Additional common EDI features included “all notion of self and identity dissolved away” (n = 134, 3.5%) and “lost all sense of ego” (n = 134, 3.5%).

### Difficult emotional responses and the Challenging Experience Questionnaire (CEQ)

Difficult emotional responses to the experience included sense of being stuck in a time loop (n = 31, 0.8%), claustrophobia or feeling trapped/lost (n = 17, 0.4%), thoughts or mind racing (n = 9, 0.2%), and suicidality (n = 9, 0.2%; see Table 9).

A total of 1019 experiences (27.0%) included a minimum of one emotional item from the CEQ (see Table 9). A total of 840 experiences (22.2%) included at least one component of the “fear” section from the CEQ, most frequently an “experience of fear” (n = 693, 18.3%). A total of 416 experiences (11.0%) included at least one component of the “grief” section from the CEQ, most frequently “emotional and/or physical suffering” (n = 386, 10.2%). A total of 107 experiences (2.8%) reported at least one component of the “insanity” section, most frequently “afraid that the state would last forever” (n = 92, 2.4%). Forty-five experiences (1.2%) reported a minimum of one item within the “isolation” subsection, most frequently “feel isolated from people and things” and “experience of isolation and loneliness” (n = 44, 1.2%, each). A total of 236 experiences (6.2%) reported at least one component of the “death” section, most frequently “feel as if dead or dying” (n = 227, 6.0%). A total of 17 experiences (0.4%) reported at least one component of the “paranoia” section.

### Statements of profundity

**Figure 3:**
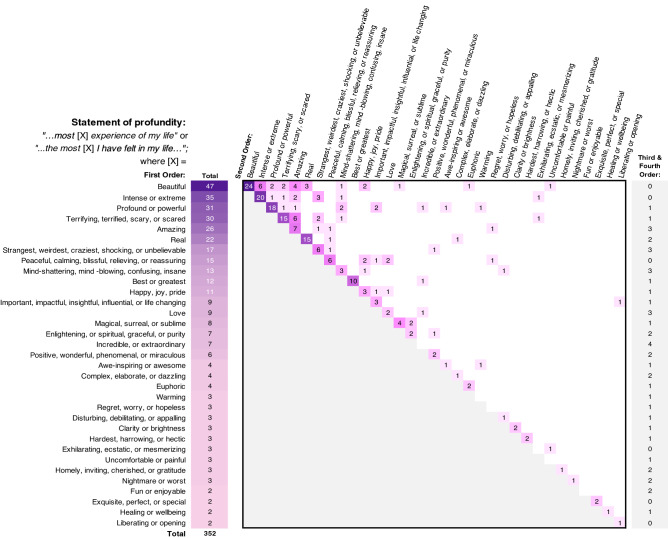
Heat map of the frequency of statements of profundity (“*…most* [X] *experience of my life…*” or “*…the most* [X] *I have felt in my life…*”, where [X] is a descriptor theme or themes). The total column represents the total coded frequency of a theme. For statements that included multiple descriptors, the order in which the themes appeared within a statement is indicated: first order column (i.e., the descriptor appeared first within the statement or alone), second order row (i.e., the descriptor appeared second within the statement), and third or fourth order column. The frequency in which first order and second order descriptors appeared jointly is highlighted at the intersection of those themes.

A total of 352 descriptors of profundity were identified in 232 experiences (6.1%, see Fig. 3). Reddit authors most frequently pronounced the experience or an aspect of the experience as the most “beautiful” or feeling the most “beautiful” of their life (n = 47, 1.2%); followed by “intense or extreme” (n = 35), “profound or powerful” (n = 31, 0.9%), “terrifying, terrified, scary, or scared” (n = 30, 0.8%), and “amazing” (n = 26, 0.7%). Statements of profundity with a single descriptor were identified in 156 experiences (4.1%).

Dual descriptors within a statement of profundity were identified in 43 experiences, of which the most common descriptors reported concurrently were “beautiful” and “intense or extreme” (n = 6); and “terrifying, terrified, scary, or scared” and “amazing” (n = 6).

## Discussion

This study conducted a qualitative analysis of naturalistic inhaled *N*, *N*-DMT experiences and documented consistent phenomenological thematic patterns, particularly within the domains of (1) physical and somatic experiences; (2) visualizations and imagery; (3) entity encounters including entity phenotype, descriptors, attributes, disposition, and characteristics of the interaction; (4) typology, architectural features, structural characteristics, and scenery of the “DMT world”; (5) alterations in consciousness (including mystical experiences, out-of-body experiences, and ego-dissolution); (6) emotional responses (including positive, rewarding, difficult, and challenging); and (7) statements of profundity.

The results of this study contribute to the limited descriptive information about inhaled *N*, *N*-DMT use and the resultant experience^11,30,43^. This study identified a reported median dose of inhaled *N*, *N*-DMT of 40 mg and duration of experience of 10 min. This is consistent with a previous study conducted in a natural setting that documented an average inhaled *N*, *N*-DMT dosage of 40 mg which induced changes in subjective and neural markers that returned to baseline after approximately 7 min^37^. Additionally, the naturalistic study conducted by Michael et al. reported a similar mean dose of inhaled DMT of 54.5 mg^30^. Somatic and physical responses to *N*, *N*-DMT administration were commonly observed in this study, most frequently the perception of somaesthesias, or kinesthetic hallucinations, and an auditory ringing sound; both of which have been previously ascribed to the DMT experience^16,19,20,26^. Gouzoulis-Mayfrank et al. documented auditory hallucinations (including “telephone rings”) in 2 out of 12 subjects (16.7%) who completed a high dose intravenous DMT session, which is a comparable incidence rate identified within this study (15.4%)^26^. Strassman et al. documented a high-pitched “whining/whirring”, “chattering,” “crinkling” sound in approximately half of the subjects at the onset of intravenous DMT administration^16^.

The features of the *N*, *N*-DMT-induced visualizations identified in this study most frequently involved the description of fractals, geometric shapes, and patterns (including descriptions of kaleidoscopes, a chrysanthemum, sacred geometry, and mandalas) and vivid, brilliant, and novel colours. Previous observations of DMT-occasioned visualizations include similar descriptions^16,19,23,26^. Strassman et al. reported that following intravenous administration of DMT “many subjects described kaleidoscopic geometric patterns that were not obviously representational”^16^, while Cott and Rock concluded that “colours, patterns, and lights were one of two common visual hallucinations”^19^. Gouzoulis-Mayfrank et al. observed that visual hallucinations consisting of “complex geometric patterns” occurred in all volunteers after receiving a high dose of intravenous DMT, although these phenomena were recorded in only a subset of volunteers following a low dose^26^. Additionally, Meyer relies on “vivid, brilliantly colored, geometric visual hallucinations” as a qualifying criterium when defining a particular “level” of DMT experience^23^.

Various theories have been proposed to explain the predictable DMT occasioned visual experience from a neurophysiologic perspective. Entoptic phenomena, defined as visual effects whose source is within the eye or visual system, can include grids or lattice patterns, parallel lines, dots or flakes, zigzags or undulating lines, nested curves, and spirals^57,58^. Entoptic phenomena featured prominently in some prehistoric art^58^ and were shown to be increased after repeated *Ayahuasca* ingestion in naturalistic settings^57^. The prevalent geometric-type visualizations identified in this study are consistent with descriptions of entoptic phenomena. Based on phenomenological studies^59^, Bressloff and colleagues developed a mathematic model suggesting that the geometrical visual hallucinations are determined by the patterns of connection between the retina, the striate cortex, and the neuronal network within the striate cortex^60^. Furthermore, results from recent electroencephalography (EEG) studies and a broader understanding of the hierarchical nature of the visual system support the empirically informed hypothesis that (1) DMT reverses the inherently top-down hierarchy of visual system and (2) DMT-visions are interpretations of propagating activity up the visual hierarchy in a bottom-up manner^14,61^. Dimethyltryptamine reproducibly (1) decreases top-down alpha-band oscillations in the cortical regions involved in visual-sensory processing, resulting in cortical excitation in these regions^14,36,61–63^, and (2) increases bottom-up forward travelling waves from occipital to frontal regions^36,61^; both of which correlate with the intensity of the DMT-induced visualizations^36,61^.

One of the most salient and discussed features of the DMT experience is encounters with seemingly autonomous entities, consistent with previous DMT studies^2,27,30^. In the current study, the most frequently reported encounters involved a feminine archetype; followed by deities, divine beings, and demi-gods; aliens, celestial beings; and extra-terrestrials; animal-based or creature-based entities (including reptilian and insectoid beings); mythological beings (including machine elves); and the jester archetype. Shanon^2^, Davis et al.^27^, and Michael et al.^30^ identified analogous entity phenotypes in their studies examining *Ayahuasca* and inhaled *N*, *N*-DMT, respectively. In the present study, communication with entities was common (75%) and comparable to rates of communication documented by Davis et al. (84%)^27^. Positive, benevolent, comforting, caring, and welcoming entity attributes and interactions were most frequently described consistent with previous reports^27^. This study provides further recognition of a “companion-type, pedagogical, or guide-type interaction” and a “medical-type” interaction^27^. Timmermann et al. predicts that the dissolution of top-down cortical control over medial temporal lobe activity may explain the content of the DMT experience, including entity encounters^17,61,64–67^.

The psychedelic disruption of top-down dynamics^17,61,64–67^ and examining psychedelic entity experiences from the perspective of evolutionary psychology and neurophenomenology has led to the theory that psychedelics may partially liberate innate modules facilitating the experience of entity encounters^5,68^. Entity encounters cannot be dismissed as irrelevant hallucinations without substance or meaning, nor accepted as transcendent realities^5^. Winkelman has identified patterns in DMT entity experiences that reflect innate operators and modules fundamental to mental processes including, but not limited to, social role inferences, agency attribution, intentionality, causality, and animacy detection^5^. These patterns suggest that DMT is, in part, exposing innate human tendencies; however, further evidence is required to substantiate any ontological claims for these entity experiences^5,69^.

Pervasive and consistent themes were reported during *N*, *N*-DMT experiences included within this study and those in other studies of the DMT-state^2^. These themes may be explained by the cultural experience and background of the authors or priming of the authors prior to the experience. Another theory to account for the pervasive themes revealed by the DMT-state is that the DMT-state is in part a probe into a collective unconscious. The collective unconscious, a term introduced by Carl Jung, consists of a repository of knowledge and imagery shared by all human beings due to a common ancestral experience^68^. It is populated with archetypes, symbols, and instincts, which greatly influence individual unconsciousness, states of mind, and behaviours^68^. Jung, however, did not provide a clear ontological account for the archetypes and the collective unconscious^70,71^. With advancements in neurophysiology and quantum physics, a theoretical approach called “biogenic structuralism” has been developed to ground Jung’s archetypal psychology within a neurophysiologic and quantum framework^70,71^. Particular attention has been placed on the relationship between the archetypes and “neurognosis”, or the understanding of how the brain develops knowledge^70^. It has been suggested that neurognosis and Jung’s archetypes are related, both, to the structural organization of the brain during development and the pattern of total quantum neural network activity^70^. Future approaches to study the DMT experience should attempt to understanding the ontological origin of prevalent themes from a neurophysiologic basis^14,61,64,65,67^.

Similarities between near death experiences (NDE) and the psychedelic experience have been described, including transcendence of physical form, themes of death and dying, OBEs, tunnels, descriptions of bright lights, familiarity, sense of belonging, religiosity, experience with a void or unearthly realm, and interaction with presences or entities^2,16,17,19,21^. These themes featured prominently within this study congruent with other studies describing the phenomenology and content of the DMT experience^2,16,17,21,27,33^. Timmermann et al. directly investigated the relatable features of the DMT experience to NDE in a within-subject placebo-controlled study of 13 healthy volunteers^17^. Significant correlations were observed between features of NDE following DMT administration compared to placebo. Moreover, a separate analysis was performed on volunteers who underwent an ‘actual’ NDE and demonstrated comparable NDE scores between an ‘actual’ NDE and the DMT state^17^. The discovery of an endogenous mammalian source of DMT^39,40^ and the similarities between the DMT state and non-drug-induced states, like NDE^17,21^, support the hypothesis that DMT is responsible for inducing certain non-ordinary states of consciousness. However, to date, it has yet to be established that endogenous DMT concentrations and selectivity are commensurate with levels required to induce effects seen during the exogenous DMT state^41,42^.

Poor recall of a DMT experience is widely reported in anecdotal sources and *N*, *N*-DMT has been described to have “a self-erasing mechanism”^72^; however, there are limited data documenting the frequency of this occurrence. The results of this study support these reports by documenting the prevalence of reported partial experience-amnesia, full experience-amnesia, and “black outs.” The amnestic mechanism of an *N*, *N*-DMT is unknown, however, may be similar to sleep dream amnesia and hypoactivation of the prefrontal cortex^73^. Alternatively, the inherent highly unusual and overwhelming nature of the DMT experience and frequent resultant ontological shock may hinder the ability of the cortex to reconstruct the experience further contributing to the reported amnestic properties of *N*, *N*-DMT.

Although many features of the DMT experience identified in this study can be labelled as positive, the presence of negative or challenging responses to DMT inhalation were also documented and important to recognize. Approximately 30% of the DMT experiences reported a minimum of one item from the CEQ; predominantly items from the “fear” subsection, followed by “grief” and “emotional and/or physical suffering” subsections. Moreover, approximately 7% of authors reported features of ego-dissolution. Ego-dissolution, defined as a compromised sense of self, can induce meaningful (i.e., a sense of unity or “oceanic boundlessness”) but also negative outcomes (i.e., “dread of ego dissolution”), with potentially psychological destabilizing effects resulting from depersonalization and derealization^55,74,75^. Overall, the negative and challenging responses to DMT ingestion should not be overlooked or underestimated. Further studies should attempt to identify individualized variables associated with a deleterious response to DMT-use. For example, this study did not capture information on concomitant mental health diagnoses, which is a critical confounder to examine in future prospective studies.

The methods within the confines of this study attempted to limit the variability of the DMT product ingested within the included experiences. Only experiences reporting *N*, *N*-DMT, as defined above, were included and experiences from other forms of DMT, including synthetic DMT and 5-MeO-DMT, were excluded. Moreover, a secondary analysis was conducted excluding experiences with reported concomitant psychoactive substance use. However, self-extraction of *N*, *N*-DMT from plant sources, such as *Mimosa hostilis* root bark and *Acacia confusa* root bark, is one of the predominant methods for obtaining *N*, *N*-DMT for naturalistic inhaled use^76^. It should be noted that the different plant sources, in addition to variable methods of extraction, can lead to variability in the concentration of the *N*, *N*-DMT and profile of other plant alkaloids in the consumed product, which may modulate the effects of the DMT^76,77^.

There are several limitations to this study. First, the naturalistic nature of not only the experience but the reporting in this study limited the ability to systematically collect data for all experience reports. This methodology allowed for the induction of novel themes and codes; however, this limits the ability to provide true incidence rates for the included variables and instead relies on rates of reported variables as a proxy for incidence. Next, Reddit users are likely nonrepresentative of DMT-users at-large and may have introduced a selection bias not controlled for in the results. Moreover, previous reports on the Reddit platform may influence the experiences and reporting of fellow Reddit users. This is evident through the recurrent invocation of specific terminology and labels for otherwise novel phenomena (i.e., “machine elves”). Reddit users are also potentially more likely to post experiences that were interesting, meaningful, consequential, and/or positive which would bias the results of this study towards the inclusion of experiences that were more notable and/or favourable. Furthermore, this bias may be especially relevant for *N*, *N*-DMT due to the reported cases of amnesic episodes identified in this study and elsewhere^27^. Adequate characterization of psychological experiences associated with *N*, *N*-DMT would require controlling for dosage and other elements that are known to influence the experience, including the set, setting, and concurrent uses of other substances. Next, the scope of the study was limited to experiences that occurred while under the influence of *N*, *N*-DMT and did not capture information pertaining to enduring effects (i.e., continuing or long-lasting). Enduring effects are valuable and can be positive or negative and should be systematically collected in future inhaled *N*, *N*-DMT studies^27^. Although this study provides insight into the naturalistic inhaled DMT-use, detailed examination in a controlled setting is required to substantiate the themes and patterns identified here.

Overall, this study contributes to the limited understanding the patterns of naturalistic inhaled *N*, *N*-DMT use and the descriptive phenomenological features of the *N*, *N*-DMT experience at-large.

## Supplementary Information

Supplementary Table 1.

Supplementary Table 2.

Supplementary Table 3.

Supplementary Table 4.

Supplementary Table 5.

## Data Availability

All requests for raw and analyzed data and materials are promptly reviewed by D.W.L (chief investigator and principal investigator). All data are anonymized and NVIVO source data with meaningful coded units are provided with this paper (see Supplemental Tables 1, 2, 3, 4, 5).
